# Complex clinical scenarios with the use of direct oral anticoagulants in patients with atrial fibrillation: a multidisciplinary expert advisory board

**DOI:** 10.1007/s12471-020-01424-y

**Published:** 2020-05-11

**Authors:** B. A. Mulder, J. ten Berg, H. ten Cate, N. van Es, M. E. W. Hemels, L. J. Kappelle, H. B. Bearda Bakker, G. J. de Borst, D. J. Drenth, G. J. Geersing, M. Rienstra

**Affiliations:** 1 Department of Cardiology, University Medical Centre Groningen, University of Groningen, Groningen, The Netherlands; 2 Department of Cardiology, St. Antonius Hospital, Nieuwegein, The Netherlands; 3 Department of Internal Medicine, Maastricht University Medical Centre, Maastricht, The Netherlands; 4 Department of Internal Medicine, University Medical Centre Amsterdam, Amsterdam, The Netherlands; 5 Department of Cardiology, Rijnstate, Arnhem, The Netherlands; 6 Department of Neurology, University Medical Centre Utrecht, Utrecht, The Netherlands; 7 Akkrum, The Netherlands; 8 Department of Vascular Surgery, University Medical Centre Utrecht, Utrecht, The Netherlands; 9 Groningen, The Netherlands; 10 Julius Centre for Health Sciences and Primary Care, University Medical Centre Utrecht, Utrecht University, Utrecht, The Netherlands

**Keywords:** Atrial fibrillation, Co-morbidities, Anticoagulation, Stroke prevention, Direct oral anticoagulant

## Abstract

The risk of developing atrial fibrillation (AF) and the risk of stroke both increase with advancing age. As such, many individuals have, or will develop, an indication for oral anticoagulation to reduce the risk of stroke. Currently, a large number of anticoagulants are available, including vitamin K antagonists, direct thrombin or factor Xa inhibitors (the last two also referred to as direct oral anticoagulants or DOACs), and different dosages are available. Of the DOACs, rivaroxaban can be obtained in the most different doses: 2.5 mg, 5 mg, 15 mg and 20 mg. Many patients develop co-morbidities and/or undergo procedures that may require the temporary combination of anticoagulation with antiplatelet therapy. In daily practice, clinicians encounter complex scenarios that are not always described in the treatment guidelines, and clear recommendations are lacking. Here, we report the outcomes of a multidisciplinary advisory board meeting, held in Utrecht (The Netherlands) on 3 June 2019, on decision making in complex clinical situations regarding the use of DOACs. The advisory board consisted of Dutch cardiovascular specialists: (interventional) cardiologist, internist, neurologist, vascular surgeon and general practitioners invited according to personal title and specific field of expertise.

## Introduction

Oral anticoagulation enables effective stroke prevention in patients with atrial fibrillation (AF) who are at risk for this severe complication. Based on the CHA_2_DS_2_-VASc score, patients with AF are advised whether or not to start anticoagulation [1]. The downside of using anticoagulation is a continuous risk of bleeding, and this risk impacts on quality of life and negatively influences drug adherence [2]. Previous AF guidelines used the HAS-BLED score to assess the risk for anticoagulation-associated bleeding [1]. However, the latest European guidelines on the management of AF did not include this particular score, but recommend evaluation of individual bleeding risk factors, and where possible treatment of the individual bleeding risk factors [1]. With the increasing number of co-morbidities and interventions in the ageing population, such as stroke, cancer and percutaneous coronary intervention or peripheral arterial interventions, complex clinical scenarios frequently arise, which require individualised decision making. Examples of complicated scenarios are: patients with stable coronary artery disease and percutaneous coronary intervention, patients with intracranial haemorrhage or recurrent bleeding in whom there is doubt about how and when to re-start anticoagulation, management of anticoagulation in those with active malignancy and AF. Most of those scenarios are not fully covered by current guidelines and clear recommendations are lacking. Medical management is further complicated by the availability of a broad spectrum of antithrombotic agents, including four different direct oral anticoagulants (DOACs). In the absence of clear recommendations, a multidisciplinary panel was instituted to discuss complex clinical situations, guided by available evidence from randomised controlled trials, post hoc analyses, and cohort and registry studies. This paper summarises the clinical situations discussed and formulates recommendations for decision making.

## Clinical situation A: chronic coronary artery disease (following percutaneous coronary intervention) and AF

### Clinical case scenario

A 62-year-old woman with known paroxysmal AF and hypertension undergoes coronary angiography for angina and documented ischaemia. The coronary angiogram shows a significant stenosis for which a percutaneous coronary intervention (PCI) is performed. What would be the best anticoagulation strategy to pursue?

### What is in the European Society of Cardiology guidelines?

**Fig. 1:**
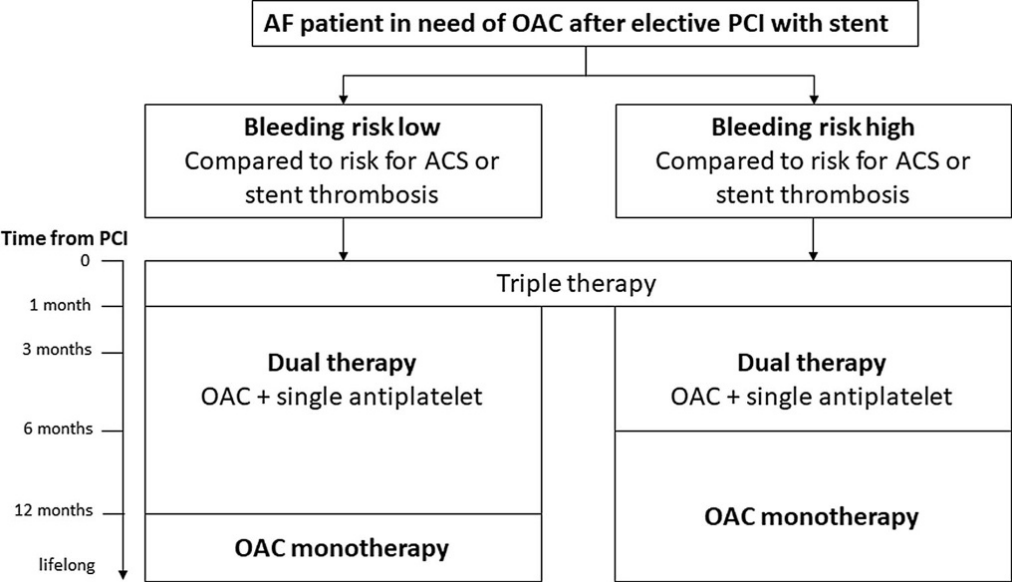
Flowchart. Atrial fibrillation (*AF*) patients and guideline recommendations after elective percutaneous coronary intervention (*PCI*). *ACS* acute coronary intervention, *OAC* oral anticoagulation. Reproduced from [1], with permission

In the most recent (2016) European Society of Cardiology (ESC) guidelines for the management of AF, a flowchart is provided for tailoring of anticoagulation after PCI in patients with AF (Fig. 1; [1]). The guideline committee, as well as our expert panel, suggest that patients with AF at risk for stroke, those with mechanical valves and those with recent or recurrent deep vein thrombosis or pulmonary embolism should continue oral anticoagulation during and after stenting [1, 3]. In general, a short period of triple therapy (oral anticoagulation, acetylsalicylic acid, P2Y_12_ inhibitor) is recommended, followed by a period of dual therapy (oral anticoagulation plus a single antiplatelet agent) [1, 3].

### Background

The ESC guidelines for the management of AF suggest that dual antiplatelet therapy (DAPT, i.e. acetylsalicylic acid + P2Y_12_ inhibitor) is indicated in patients using oral anticoagulation in whom a PCI is performed. However, triple therapy (DAPT plus oral anticoagulation) is associated with a significantly increased risk of major bleeding. For example, the WOEST trial demonstrated that an approach *without* acetylsalicylic acid—in that trial a vitamin K antagonist (VKA) combined with clopidogrel significantly reduces bleeding risk during follow-up (19% for patients with dual therapy compared to 44% for those with triple therapy) [4]. However, when this trial was conducted, DOACs had not yet been introduced in the clinical setting. The effectiveness and safety of anticoagulation with the use of a DOAC (rivaroxaban) combined with acetylsalicylic acid or DAPT was compared with VKAs in the PIONEER AF-PCI study [5]. The study’s main conclusion was that the administration of either low-dose rivaroxaban (15 mg o.d.) plus a P2Y_12_ inhibitor for 12 months or very-low-dose rivaroxaban (2.5 mg b.i. d.) plus DAPT for 1, 6, or 12 months was associated with a lower rate of clinically significant bleeding compared to standard therapy with a VKA (16.8% vs 18.0% vs 26.7%, respectively) plus DAPT for 1, 6, or 12 months [5]. Although the study was not powered to assess the outcomes of survival, myocardial infarction and stroke, no difference was observed in the incidence of these events among the three groups. Notably, the occurrence of stroke was very low (6.5% vs 5.6% vs 6.0%, respectively), although this is partly explained by the exclusion of patients with a history of stroke or transient ischaemic attack. In the original ROCKET AF trial the lower dose of rivaroxaban (15 mg o.d.) was used only in patients with moderate kidney disease (creatinine clearance 30–49 ml/min) [6, 7]. However, when observing registry data, it appears that the 15-mg dose is often prescribed in clinical practice, even in those patients with normal kidney function [8]. It is unclear why this choice is made by treating physicians, but it is likely due to a perceived feeling or the actual presence of risk factors for bleeding, including advanced age and relevant co-morbidities. Importantly, one registry showed that the use of reduced doses of apixaban (2.5 mg b.i. d.) or rivaroxaban (15 mg o.d.) was associated with an increased risk of death, compared to warfarin, emphasising the need to prescribe the correct dosage for each DOAC [9]. The RE-DUAL PCI study randomised 2725 patients with AF and a PCI to either triple therapy (dabigatran 110 or 150 mg b.i. d., acetylsalicylic acid and warfarin) or dual therapy consisting of dabigatran 110 or 150 mg b.i. d. plus a P2Y_12_ inhibitor (clopidogrel or ticagrelor) and no acetylsalicylic acid. Dual therapy was non-inferior to triple therapy with respect to the risk of thromboembolic events [10]. Also, in the AUGUSTUS trial of patients with AF undergoing a PCI, the combination of a P2Y_12_ inhibitor with full-dose apixaban resulted in less bleeding and fewer hospitalisations without significant differences in the incidence of ischaemic events compared to regimens that included a VKA, acetylsalicylic acid, or both [11]. Finally, these findings are also confirmed in the edoxaban-based versus VKA-based antithrombotic regimen after successful coronary stenting in patients with AF (ENTRUST-AF PCI) for the use of edoxaban as compared with VKA [12]. A recent meta-analysis of the aforementioned studies confirmed the finding that dual therapy with a DOAC and P2Y_12_ inhibitor led to a reduction in major and intracranial bleedings. However, a higher risk of cardiac (mainly stent-related) ischaemic occurrences was observed [13]. Therefore in the case of a high ischaemic risk (e.g. acute clinical presentation, or certain difficult anatomical or procedural features), as recommended by the ESC revascularisation guidelines, triple therapy may be continued for a longer period of time (6 months) [14]. A longer duration of triple therapy is also often required in patients with acute coronary syndromes; however, these patients were beyond the scope of this article, and detailed recommendations may be found in the respective ESC guidelines [15, 16]. For example, for a patient admitted with a non-ST-elevation acute coronary syndrome and a low to intermediate bleeding risk (as assessed by the HAS-BLED score) triple therapy is recommended for 6 months. In the case of a high bleeding risk the period of triple therapy is reduced to 4 weeks [15].

### Consensus of advisory board

The expert panel opinion was that the optimal approach for patients with AF who have undergone PCI should be *dual therapy* and the full permissible dosage of any DOAC. There was also consensus that in the case of rivaroxaban the choice of a lower dosage is to be considered based on the findings of the PIONEER AF-PC trial, but only in patients without a history of stroke or transient ischaemic attack. For the other DOACs, no data are available about the efficacy of lower doses or their safety as regards stroke prevention. Nonetheless, when a patient is considered to be at a higher risk for bleeding (for any reason) that outweighs the stroke risk, a lower dose of a DOAC seems reasonable. Conversely, in a patient at low risk for bleeding, a full dose of a DOAC should be the standard approach, depending obviously on kidney function.

## Clinical situation B: stable lower extremity arterial disease and AF

### Clinical case scenario

A 56-year-old man with AF without co-morbidities is seen by the vascular surgeon at the outpatient clinic. The surgeon diagnoses the patient with symptomatic peripheral artery disease (PAD) and consults the treating cardiologist regarding the consequences of this diagnosis for antithrombotic management. What would be the best anticoagulation strategy to pursue for this patient?

### What is in the ESC guidelines?

**Fig. 2:**
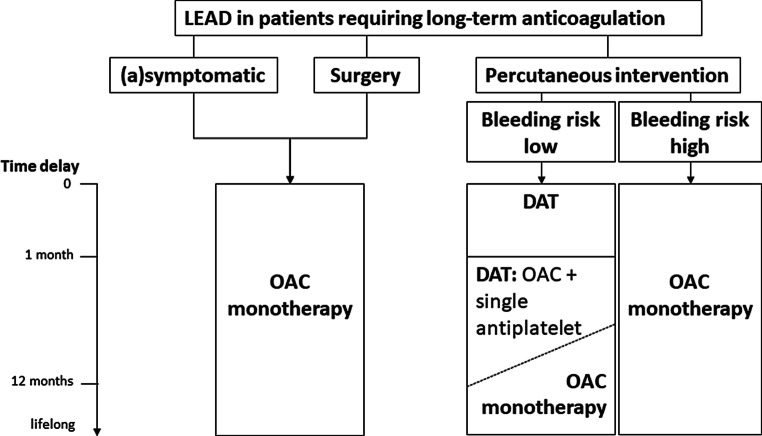
Flowchart. Lower extremity arterial disease/peripheral artery disease (*LEAD*/*PAD*) and AF. *DAT* dual antithrombotic therapy, *OAC* oral anticoagulation. Reproduced from [17], with permission

The 2017 ESC guideline on the diagnosis and treatment of PAD includes a section on patients with AF (Fig. 2; [17]). Except for recent peripheral artery stenting, patients with PAD and AF should mostly remain on oral anticoagulation alone, without the addition of antiplatelet therapy. No specific recommendation regarding DOACs (or dosages) is made in this guideline.

### Background

The CHA_2_DS_2_-VASc score used in all patients with AF to assess stroke risk includes vascular disease as a component [18]. Hence, patients with AF and a history of PAD have an indication for the AF dose of a DOAC [1]. The most recent PAD guideline of the ESC was released in 2017 [17]. The flowchart presented in this guideline shows that in a(*n*) (a)symptomatic patient with AF and PAD who requires long-term anticoagulation it is sufficient to continue oral anticoagulation monotherapy [17].

### Consensus of advisory board

The suggestion of this advisory board is that in a (highly) symptomatic PAD patient, despite intensive walking therapy, addition of a single antiplatelet therapy might be considered with background therapy with a DOAC, in particular in patients in whom revascularisation is performed. The duration of such dual therapy should be balanced with bleeding risk and preferably be as short as clinically feasible. Nevertheless, this certainly is an area where more research is greatly needed. For instance, the recently published AFIRE trial (addition of antiplatelet therapy to rivaroxaban versus rivaroxaban monotherapy in patients with AF and stable coronary artery disease) was stopped prematurely because of an increased mortality rate in patients treated with dual therapy [19]. In conclusion, in patients with AF and stable PAD full-dose monotherapy with a DOAC is a reasonable alternative to a VKA in most patients.

## Clinical situation C: stroke in patients with AF

### Clinical case scenario

A 76-year-old women with AF (CHA_2_DS_2_-VASc score of 5) who is using a DOAC for stroke prevention is admitted to the emergency department with stroke-like symptoms. After performing a CT scan, the diagnosis of a parenchymal haemorrhagic stroke is made by the attending neurologist. Assuming the patient survives this critical bleeding, what is the recommendation for anticoagulation use after this complication?

### What is in the ESC guidelines?

The AF guidelines do not provide any specific recommendations on the use of DOACs after a haemorrhagic stroke [1]. There is a general recommendation suggesting that re-initiation of oral anticoagulation after a bleeding event should be considered in all eligible patients by a multidisciplinary team, considering different anticoagulants and stroke prevention interventions [1].

### Background

The risk of stroke in AF patients is considerable despite anticoagulant treatment. In the case of ischaemic stroke, current management foresees the use of catheter-guided clot removal, often in association with thrombolytic treatment. In patients receiving anticoagulation, the use of thrombolytic agents is, however, not deemed safe. Only in the absence of detectable anticoagulant activity in blood, such as a low INR in patients on a VKA, is thrombolytic therapy considered. For DOACs, this remains a challenge in the absence of a good point-of-care test (except for dabigatran, which can be ruled out with a normal thrombin clotting time) [20]. However, it is generally considered that when a patient has normal renal function and the last dose was more than 24 h ago there is no contra-indication. In a post hoc analysis of the ROCKET AF study of 19 patients receiving thrombolytic therapy, mainly for ischaemic stroke, it was shown that non-fatal major bleeding and death occurred in 2 and 2 patients, respectively. These events mostly occurred when thrombolytic therapy was administered within 48 h of the last rivaroxaban dose [21]. Of the 9 patients in the warfarin group, 1 experienced a non-fatal major bleeding event and 3 died, most occurring when thrombolytic therapy was administered more than 48 h after the last warfarin dose. This study suggests that careful assessment of the time since the last dose may be of clinical significance in patients on DOACs who require emergent thrombolysis. In addition, some routine laboratory tests can be used to exclude the presence of significant levels of a DOAC, such as the diluted thrombin time, which is highly sensitive for dabigatran [22]. Another theoretical possibility is the use of a DOAC antidote before thrombolysis, although there is currently little support for this practice among neurologists.

Current recommendations on (re-) starting oral anticoagulation *after* acute ischaemic stroke must weigh (recurrent) stroke risk against secondary haemorrhagic transformation. After an acute ischaemic stroke it is probably safe to re-start anticoagulation after 1–14 days, depending on bleeding risk factors, including the use of thrombolytic agents [23, 24].

A recent meta-analysis showed that resuming anticoagulation 4–8 weeks after an intracranial bleed is associated with fewer ischaemic strokes and that there is no increased risk of re-bleeding [25, 26]. Currently, a Dutch trial is including patients with a history of AF and a recent intracerebral haemorrhage during treatment with anticoagulation (APACHE-AF, clinicaltrials.gov: NCT02565693). These patients are randomly assigned in a 1:1 ratio to either apixaban or no oral anticoagulation.

### Consensus of advisory board

The consensus is that a full dosage of a DOAC can be re-initiated 14 days after an ischaemic stroke, provided that any risk factors for bleeding are not persistent but manageable [27]. In the case of haemorrhagic stroke, anticoagulation could be re-initiated after 4–8 weeks after careful consideration of the pros and cons of anticoagulation, including type and dosage.

## Clinical situation D: patients who have a malignancy and AF

### Clinical case scenario

A 55-year-old man with AF, hypertension and diabetes mellitus has been diagnosed with gastric cancer. He is admitted to the emergency department with haematemesis for which urgent endoscopy is indicated. The attending gastroenterologist calls to discuss the patient’s active DOAC use. What are the options for this patient?

### What is in the ESC guidelines?

Malignancy is considered a risk factor for bleeding in the latest ESC guidelines on the management of AF [1]. Nevertheless, there are no clear recommendations in the guideline with regard to the choice of a specific type of oral anticoagulant for patients with an active malignancy. The ESC guidelines do, however, recommend a cautious approach when using DOACs in combination with systemic anticancer therapies that influence CYP3A4 or P‑glycoprotein to avoid under- or overdosing.

### Background

If a patient with AF develops cancer, clinicians should carefully re-assess the risks of stroke versus bleeding. In general, there are several options with regard to the use of anticoagulation: continue anticoagulation unchanged, switch from a DOAC to low-molecular-weight heparin, reduce the dose of a DOAC or (temporarily) discontinue oral anticoagulation in patients deemed at high risk for bleeding, or stop anticoagulation permanently in the case of a short life expectancy. One of the main issues of anticoagulation in patients with a malignancy and DOAC is that the risks of thromboembolism and bleeding vary widely across tumour types, which complicates a ‘one size fits all’ approach. Overall, in a prospective study, it was shown that the AF-related thromboembolic risk is not influenced by cancer status, but that patients with active cancer do have an increased risk of bleeding [28]. These bleeding events can be related to the tumour itself (e.g. gastrointestinal, urinary tract, or brain), diagnostic or therapeutic interventions, increased anticoagulant drug levels due to kidney injury (as a consequence of hypovolaemia or nephrotoxic drugs), thrombocytopenia (e.g. due to chemotherapy-induced bone marrow suppression), cancer treatment (e.g. ibrutinib or bevacizumab), or due to interaction with concomitant CYP3A4 or P‑glycoprotein inhibitors. However, in a post hoc analysis of the ROCKET AF trial, it was shown that the safety and efficacy of rivaroxaban treatment for AF in patients with active cancer are comparable with the results of the ROCKET AF study in the general population (albeit that particular subtypes of cancer, e.g. gastric cancer, may yield a higher risk for bleeding) [29]. Also a subanalysis of the ENGAGE AF-TIMI 48 trial showed that in patients with AF who develop malignancy, the efficacy and safety profile of edoxaban relative to warfarin is preserved [30]. Patients with a gastrointestinal malignancy have a higher risk for anticoagulation-related bleeding, especially when using a DOAC [31].

### Consensus of advisory board

It is the opinion of the advisory board that in general anticoagulation should be continued unchanged in patients with AF and cancer. Temporarily switching from oral anticoagulation to low-molecular-weight heparin is an alternative when patients are unable to orally ingest drugs (e.g. due to nausea) or in the presence of a significant drug-drug interaction. Furthermore, a DOAC is not preferred in patients with a luminal gastrointestinal cancer, and possibly also not in patients with urogenital cancer (although in this case there is only limited data). In such patients, switching from DOAC treatment to a reasonable alternative, such as a VKA or low-molecular-weight heparin, should be considered. Finally, anticoagulant therapy should generally be discontinued in the case of severe thrombocytopenia (<50 × 10^9^/l, or consider platelet transfusion), abnormal liver function, or severe kidney injury (creatinine clearance <30 ml/min); however, then a VKA is still an option.

## Conclusions

**Fig. 3:**
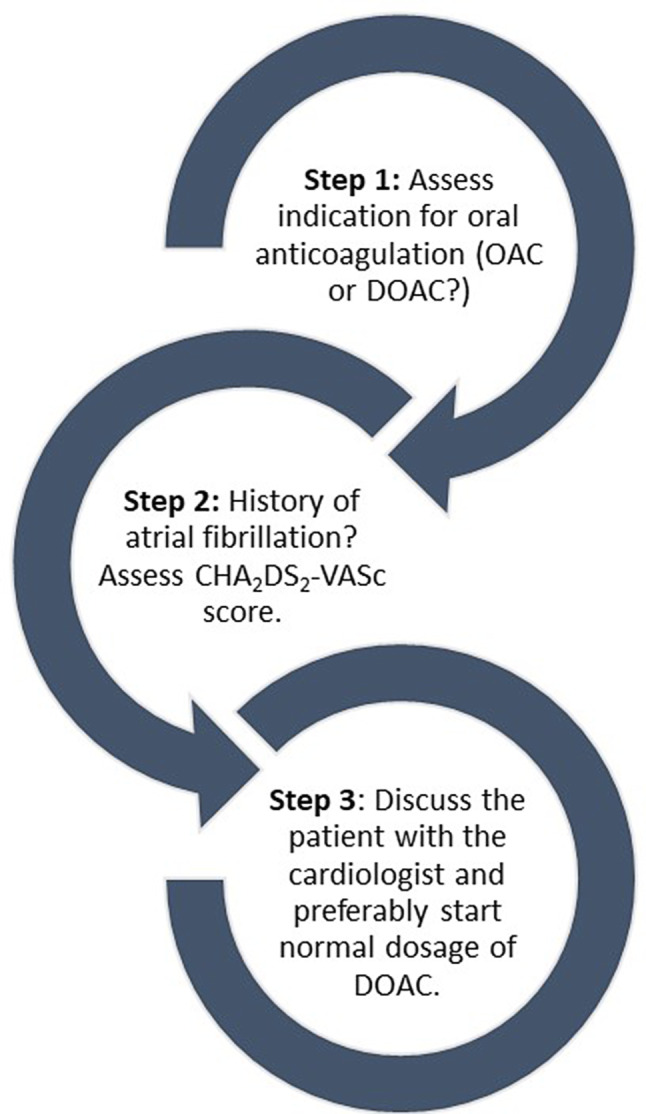
Framework for the initiation of direct oral anticoagulant (*DOAC*). *OAC* oral anticoagulant

In this article, by providing four case scenarios we tried to summarise the evidence for managing several major dilemmas we often encounter in clinical practice and for which current guidelines on antithrombotic therapy do not provide clear recommendations, or where new data has been published recently. Fig. 3 shows a framework which may help in decision making in the clinical setting. It is important to remember that our advice is to use a full-dose regimen of a DOAC if possible, as this is the dosage investigated in the major DOAC trials, but that the dosage may need to be individualised under specific circumstances, albeit there are no supporting data. Resuming anticoagulation is in general recommended, provided there are no persistent risk factors for bleeding that exceed the risk of thromboembolism.
